# Changes in Somatomotor Network Connectivity Differentiate Better Versus Worse Recovery of Hand Dexterity After Brain Tumor Surgery: A Cohort Study

**DOI:** 10.1155/np/3255120

**Published:** 2026-09-21

**Authors:** Timothy F. Boerger, Leon Taquet, Kaitlin Goetschel, Sarah C. Young, Jennifer Connelly, Jeffrey R. Binder, Brian D. Schmit, Max O. Krucoff

**Affiliations:** ^1^ Department of Neurosurgery, Medical College of Wisconsin, Milwaukee, Wisconsin, USA, mcw.edu; ^2^ Joint Department of Biomedical Engineering, Marquette University and Medical College of Wisconsin, Milwaukee, Wisconsin, USA; ^3^ Department of Global Health Equity, Medical College of Wisconsin, Milwaukee, Wisconsin, USA, mcw.edu; ^4^ Department of Neurology, Medical College of Wisconsin, Milwaukee, Wisconsin, USA, mcw.edu; ^5^ Department of Biophysics, Medical College of Wisconsin, Milwaukee, Wisconsin, USA, mcw.edu

**Keywords:** brain tumor, dexterity, functional connectivity, recovery

## Abstract

**Introduction:**

Hand dexterity is critical for everyday function and involves the coordinated integration of multiple brain networks. However, we know very little about how changes in connectivity across networks facilitate recovery after removal of a pathological brain lesion, like a tumor.

**Methods:**

Resting‐state functional connectivity and 9‐hole peg test (9HPT) times were collected before and after surgical resection in 16 patients with brain tumors. All patients had contrast‐enhancing brain lesions (12 glioblastomas, 2 metastases, and 2 radionecrosis). Patients were retrospectively grouped into better or worse recovery based on change in 9HPT scores. Connectivity was assessed between the ipsilesional dorsomedial somatomotor network A (SMN A) with 39 other networks in the brain using partial correlations.

**Results:**

Patients with worse recovery of hand function had a decrease in somatomotor connectivity to the rest of the brain (*p*  < 0.05) and a decrease in interhemispheric connectivity to the contralesional SMN A (*p*  < 0.05). Conversely, patients with better recovery had higher ipsilesional SMN A to ventrolateral SMN B connectivity preresection (*p*  < 0.05) and higher ipsilesional SMN A‐to‐contralesional dorsal attention network B (DAN B) connectivity postresection (*p*  < 0.05).

**Conclusions:**

Recovery of hand dexterity from presentation to postoperative follow‐up in patients with brain tumors most strongly correlated with ipsilesional SMN A connectivity to the contralesional SMN A, contralesional DAN B, and ipsilesional SMN B. This finding suggests promising neuromodulation targets for postoperative therapies aiming to improve hand dexterity.

## 1. Introduction

The goal of the present study was to define the relationship between recovery of resting‐state functional connectivity and hand dexterity in patients with brain tumors who underwent surgical resections to guide future therapies seeking to maximize neurological outcomes. Brain tumors can induce motor deficits by infiltrating, compressing, or spreading edema into critical motor areas [1, 2]. While injury to primary motor cortex and/or its descending corticospinal tracts results in limb paralysis, functional–anatomical relationships subserving higher‐order motor functions, like hand dexterity, involve the integration of multiple neurological domains, are less easily localized, and are more likely to be impacted by larger‐scale brain network connectivity [2–6]. Because sensorimotor deficits are associated with poorer overall survival and worse quality of life [7], interventions that improve motor functions could improve both [8]. To advance the currently limited arsenal of therapeutic options, a better understanding of natural cortical reorganization differentiating successful from failed recovery is necessary.

Connectivity between the sensorimotor network and the rest of the brain (termed “node strength”) has been associated with motor impairments [4, 9]. Likewise, reduced interhemispheric connectivity in patients with brain tumors compared to healthy controls has also been prospectively and independently associated with worse survival [10]. Further, there is evidence that within‐hemisphere functional reorganization can occur due to either the presence or resection of brain tumors [11, 12]. For example, in one study, ipsilesional motor activation magnitude during task‐based functional magnetic resonance imaging (fMRI) decreased following resection of brain tumors [13]. Additionally, other studies have shown that both transcranial magnetic stimulation‐based and intraoperative stimulation‐based motor maps can spatially shift over time, implying reorganization of functional–anatomical relationships within the somatomotor network (SMN) [12, 14–19]. Finally, we have even reported a case where the primary motor cortex was functionally localized outside of the anticipated precentral gyrus at an initial surgical resection of a motor area metastasis in an adult [11]. Outside of this collection of small case series and reports, longitudinal, quantitative assessments of sensorimotor control and functional brain network reorganization are scarce for patients recovering from brain tumor surgery.

Therefore, to address this gap, here, we conducted a longitudinal observational study examining changes in both dexterity and functional brain‐network connectivity from presurgery to postrecovery follow‐up. Our overall objective was to better understand which changes in resting‐state fMRI‐based functional connectivity were associated with improvements versus declines in dexterity to inform future targeted therapeutic neuromodulation studies [20–25].

## 2. Methods

This is a longitudinal cohort study of functional connectivity based on resting‐state fMRI, with the dependent variable as improvement in 9‐hole peg test (9HPT) time from presentation (preoperative) to postoperative follow‐up (median 4‐months postoperative, range 3–8.4 months). Recruitment occurred from October 2022 to December 2024 at a tertiary care hospital. Participants were retrospectively separated (nonrandomly) into two groups based on whether their reduction in (9HPT) time for the contralesional hand was larger (better recovery) or smaller (worse recovery) than the median. For example, as the median reduction in 9HPT time was 4.24 s, a participant whose 9HPT time improved by 5 s would be placed into the better recovery group. We used this method because (1) the data were strongly skewed due to participants unable to perform the task, (2) there were three outlier responses (isoutlier; MATLAB, Natick, MA) who had substantial improvements or worsening of performance, (3) we desired the interpretation to be both clinically and scientifically meaningful, and (4) to follow precedent in using dichotomized continuous outcome and predictor data to understand changes in brain function [26–30]. Secondarily, we additionally analyzed the dexterity data in continuous form, as described below in Section 2.6 Statistical Analysis. The 9HPT is a validated measure of hand dexterity [31]. Secondarily, we examined whether connectomic changes might be associated with several known, clinically relevant covariates such as proximity to motor regions, age, biological sex, glioblastoma versus non‐glioblastoma, O‐6‐methylguanine‐DNA methyltransferase methylation status, and isocitrate dehydrogenase wild type. This study was approved by the Institutional Review Board at the Medical College of Wisconsin and conformed to the Declaration of Helsinki.

### 2.1. Participants

Demographic data other than tumor measures and 9HPT were extracted from the patient’s medical record. Inclusion criteria required the patient to have a planned resection for a contrast‐enhancing lesion and a clinical need for an fMRI for surgical planning. Our target sample size was chosen based on its similarity to other studies. Exclusion criteria included prior brain surgery regardless of whether that surgery was for a tumor or not. Additionally, participants were excluded if they had a history of other neurological diseases that may impact upper extremity function, such as demyelinating disease or cervical myelo‐ or radiculopathy. The diagnosis of probable contrast‐enhancing brain tumor was made based on standard imaging features and was pathologically confirmed from surgical biopsies. Notably, in one case, the diagnosis after biopsy was changed to ischemic stroke, and this participant was withdrawn from the study. Additionally, in two cases, the diagnosis after biopsy was radionecrosis. These participants were retained as radionecrosis can impact both connectivity and brain function.

A number of clinical and demographic variables unique to brain tumors were collected in this study. Different contrast‐enhancing lesion types (glioblastoma [i.e., high‐grade primary brain cancer] vs. metastasis [most common primary cancers in literature are from lung, breast, and melanoma] vs. radionecrosis [necrotic tissue due to pre‐surgical treatment with radiosurgery]) have differing growth and infiltrative patterns that could impact brain function and therefore, recovery.

### 2.2. Hand Motor Function Testing

9HPT scores were obtained for both the left and right hands independently prior to surgery and again at postoperative follow‐up. The 9HPT is a measure whereby participants pick up pegs individually and place them into a hole and then remove them as quickly as possible [31]. It was originally used as a measure of recovery of dexterity in stroke but has more recently been used in brain tumors and has been adopted as a general measure of hand dexterity for the NIH [32–34]. The participants were given verbal encouragement. Time was capped at 120 s per convention. Two trials were collected for each hand, and the second score of the contralesional hand was used for analysis, as per convention [34]. 9HPT data are presented in raw (s) and population‐normalized (*z*‐scores) formats, as per previously published norms [35].

### 2.3. Image Acquisition

High‐resolution pre‐ and postcontrast T1 as well as fluid attenuate inversion recovery (FLAIR) and standard T2 images were obtained as part of the patient’s standard of care presurgical workup. Additionally, clinical resting‐state fMRI scans were obtained for surgical planning. All presurgery images were obtained primarily for clinical care. As such, fMRI parameters varied based on the clinical protocol but ranged from 7–10 min, time repetition/time echo: 2–3.157/0.03s, matrix: 64 × 64–80 × 80, slice thickness: 3–5 mm, and flip angle = 90. Variances in scan parameters were accounted for statistically and they did not significantly account for differences in variables of interest for functional connectivity (*p*  > 0.20). All scans were conducted on a GE (Waukesha, WI) Signa 3.0T scanner with a 48‐channel head coil. Parameters for the resting‐state fMRI scans obtained for research purposes at postoperative follow‐up were set to match the individual’s presurgical scan.

### 2.4. Image Analysis

Anatomical images (T1, T1C, FLAIR, and T2) were used for tumor segmentation using the Cancer Imaging Phenomics Toolkit [36]. T1 images were used to register the participants’ brains to the Montreal Neurological Institute 2 mm standard space using Advanced Normalization Tools software [37]. Tumor masks were used to find the minimum Euclidean distance (MATLAB [Natick, MA]; *pdist2*) between any voxel occupied by the tumor and the hand knob and the corticospinal tract. This was done to evaluate any effect the distance between the tumor and the motor system may have had on recovery. Hand knob locations were defined from regions of interest (ROI) extracted from the Schaefer 2018 brain atlas [38]. The location of the corticospinal tract was defined based on the Johns Hopkins white matter atlas [39], available in the FMRI Software Library (FSL, Oxford, UK).

**Figure 1:**
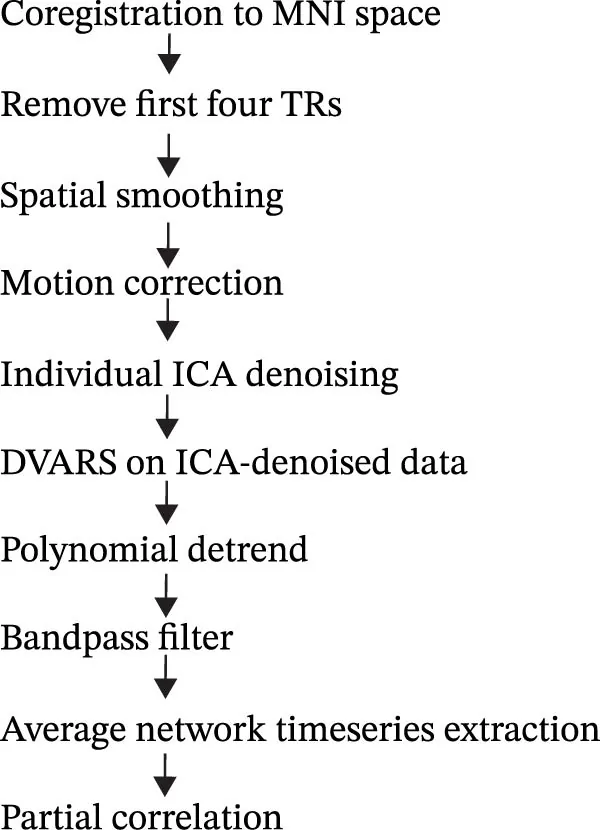
fMRI data processing flowchart.

The processing workflow for calculating functional connectivity is displayed in Figure 1 as we used previously [40]. All images were brain‐extracted and bias‐corrected. Functional images were additionally processed by deleting the first four volumes, spatial smoothing, motion correction (FSL: MCFLIRT), and independent component analysis denoising (ICA‐AROMA) [41]. Residual noise was identified using the difference variance (DVARS) [42] algorithm in FSL (fsl_motion_outliers) to identify image volumes that were >75^th^ percentile + 1.5x the interquartile range of DVARS values for an individual [43]. Censoring with interpolation, based on DVARS‐defined outlier volumes, was conducted using analysis of functional neuroimages (AFNI) [44] 3dTproject along with polynomial detrending and bandpass filtering between 0.01 and 0.08 Hz. After processing, images of participants with right‐side tumors were flipped right to the left. Network timeseries were extracted using the Yeo 2011 atlas [45], which was split into left and right hemispheres. ROIs for the left and right‐side cerebellum, basal ganglia, and thalamus were appended to the atlas from FSL’s built‐in atlases derived from prior studies [46–48]. Similar methods of appending subcortical regions to cortical atlases have been previously performed [49]. Partial correlation (FSLNets toolbox; using the L1‐norm regularization) was used to calculate functional connectivity between every pair of networks accounting for the connectivity of every other network [50]. Fisher’s *r* to *z* transformation was then performed.

### 2.5. Network Connections of Interest

We focused on the following three connections that we anticipated would correlate with recovery based on data from prior studies explored above: (1) SMN to the rest of the brain, (2) SMN to SMN, and (3) SMN to the dorsal attention network (DAN). Secondarily, our analyses examined if connectomic changes were more associated with several known clinically relevant covariates, which are located in the Supporting Information 1.

**Figure 2:**
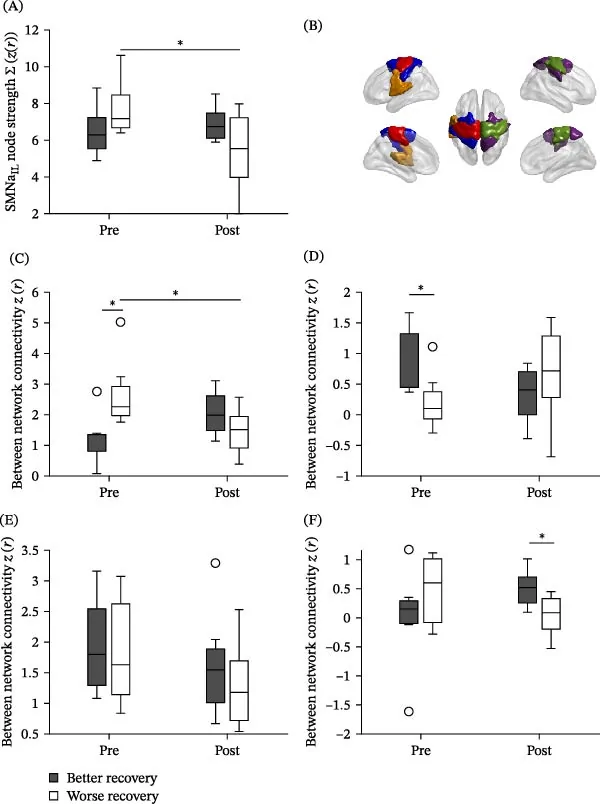
Between network connectivity results. (A) SMN *A*_IL_ node strength. (B) Topography of networks of interest for pairwise connectivity analyses used in C–F. Red = SMN *A*_IL,_ blue = DANb_IL_, green = SMN *A*_CL_, gold = SMN *B*_IL_, and purple = DAN *B*_CL_. (C) SMN *A*_IL_–SMN *A*_CL_ homologous interhemispheric connectivity. (D) SMN *A*_IL_–SMN *B*_IL_ heterologous intrahemispheric connectivity. (E) SMN *A*_IL_–DAN B_IL_ heterologous intrahemispheric connectivity. (F) SMN *A*_IL_–DAN *B*_CL_ heterologous interhemispheric connectivity.  ^∗^ *p* < 0.05.

The spatial map of the Yeo atlas [45] whole brain network coverage we used is presented in Figure 2 for reference. For this study, we were most interested in networks associated with sensorimotor functions, motor planning, and visual spatial attention, including the SMN A, SMN B, and DAN B, whereas the DAN A network is topographically associated with visual association areas and therefore of less interest in this study [45, 51]. The SMN A is largely composed of Brodmann’s areas 4, 3, 1, and 2, and it extends from the medial aspect of the cerebrum to just beyond the anatomical hand knob laterally as well as containing a posterior portion of the support motor area. The SMN B, that is, “lateral SMN” described in the introduction, includes the facial portion of the motor and sensory cortices (areas 4, 3, 1, and 2), as well as the superior aspects of the temporal lobe involved in auditory processing. DAN B incorporates areas such as the premotor cortex, frontal eye fields, and posterior parietal cortex. Primary connections of interest representing our *a* priori hypothesis outlined above were: (1) sum of ipsilesional SMN A to the rest of the networks of the brain (e.g., node strength) [52], (2) the ipsilesional SMN A to the contralesional SMN A, (3) ipsilesional SMN A to the contralesional DAN B, (4) ipsilesional SMN A to the ipsilesional SMN B, and (5) ipsilesional SMN A to the ipsilesional DAN B.

### 2.6. Statistical Analysis

Statistical analyses were conducted in SPSS (IBM Inc., Armonk, New York). Mixed model analyses of covariance (ANOVA) were conducted to compare the outcome group (i.e., better recovery vs. worse recovery; between‐participants independent variable) by time (within‐participants independent variable) with connectivity for our previously mentioned connections of interest (dependent variables). *p*‐Values were Sidak‐corrected. Effect sizes for pairwise comparisons were calculated using Cohen’s *d*. Shapiro–Wilk, Box’s, and Levene’s tests were conducted to ensure that assumptions of normality and homogeneity of variance were not violated. Fischer’s exact chi‐square test, *T*‐tests, and Mann–Whitney *U* tests (as appropriate based on data distributions) were conducted to determine differences between outcome groups at baseline. Parametric linear regressions were used to determine demographic predictors of change in connectivity (defined as follow‐up connectivity minus presurgical connectivity). Potential demographic predictors were left‐sided lesion, tumor volume, tumor proximity to the hand knob, proximity to the SMN, proximity to the corticospinal tract, right‐handedness, tumor in the hemisphere opposite the dominant hand, extent of resection (gross total vs. near total vs. subtotal), age, biological sex, glioblastoma versus non‐glioblastoma, O‐6‐methylguanine‐DNA methyltransferase methylation status, isocitrate dehydrogenase wild type, and baseline dexterity. Alpha was set at *p*  < 0.05. Loss to follow‐up was handled with case‐wise deletion.

Secondarily, to analyze the connectomic relationship with dexterity in a continuous format, we performed linear mixed‐effects models in *R* (R Foundation for Statistical Computing, Vienna, Austria). *R* libraries for analysis and visualization included lme4, car, psych, lmersampler, sjPlot, and patchwork. Linear mixed‐effects models were bootstrapped with 100,000 resamples. *p*‐Values were extracted from bootstrap distributions and adjusted for the false discovery rate using the Benjamini–Hochberg method. Prior to statistical analyses, data were *Z*‐normalized using published normative data [35], shifted to have a minimum >0, and log_10_‐transformed to address the positive skew. In *R*, all data were then *z*‐scaled prior to the statistical modeling. Statistical methods and results of an additional data‐driven analysis are included in the Supporting Information 1.

## 3. Results

### 3.1. Demographic and Tumor Variables

Thirty‐two participants were initially enrolled in the study and completed presurgical hand motor testing and functional imaging. Of these, 16 patients completed follow‐up motor testing and functional imaging. One participant was withdrawn due to a pathological diagnosis of ischemic stroke, and another participant withdrew prior to baseline testing for personal reasons. Three participants died prior to follow‐up testing. The remaining 11 participants declined to continue participation due to the intensity of the ongoing clinical treatment. Demographic data from our longitudinal cohort who completed both baseline and follow‐up testing is presented in Table 1. There were no differences in any of the demographic variables between patients with a better or worse outcome based on the 9HPT; however, age approached statistical significance (independent sample *t*‐test; *p* = 0.05). In our cohort, 12 participants had glioblastomas, two had metastases, and two had radionecrosis from prior radiosurgery. The primary cancers of the two participants with metastases were of urothelial and renal origins, whereas the primary cancer of both participants with radionecrosis was non‐small‐cell lung cancer. Three total participants had new intraoperative sensorimotor changes. Of these, one participant had a clinically defined intraoperative sensory deficit during awake surgery. Another participant had a reduction in intraoperative somatosensory evoked potentials. The third participant had a reduction in motor‐evoked potentials. Intraoperative sensorimotor changes did not significantly correlate with dexterity outcome (*p*  > 0.05), suggesting our results were unlikely to be driven by these changes. Dexterity scores were normalized to healthy population data such that higher scores represented worse dexterity (*z* = 7.78 ± 11.43 preoperatively and *z* = 6.12 ± 12.19 postoperatively). Table 1 reports the change scores from the contralesional hand 9HPT used to binarize our cohort.

**Table 1:** Demographic data.

	Total (*n* = 16)		Better		Worse		*p*‐Value
Male, *n* (%)	9	(56.25)	4	(50.00)	5	(62.50)	>0.05
Age, mean (sd)	59.25	(11.68)	64.88	(8.79)	53.63	(11.94)	0.05
Glioblastoma, *n* (%)	12	(75.00)	6	(75.00)	6	(75.00)	>0.05
Metastasis	2	(12.50)	2	(25.00)	0	(0.00)	>0.05
Radionecrosis	2	(12.50)	0	(0.00)	2	(25.00)	>0.05
Methylguanine‐DNA methyltransferase gene promotor methylated, *n* (%)	7	(43.75)	3	(37.50)	4	(50.00)	>0.05
Isocitrate dehydrogenase wild type, *n* (%)	10	(62.50)	6	(75.00)	6	(75.00)	>0.05
Postoperative restricted diffusion, *n* (%)	11	(68.75)	7	(87.50)	4	(50.00)	>0.05
Follow‐up months, mean (sd)	4.98	(1.97)	5.41	(2.26)	4.54	(1.67)	>0.05
Tumor volume, mean (sd)	31.28	(25.10)	41.39	(27.97)	21.16	(18.26)	>0.05
Flair volume, mean (sd)	54.48	38.91	63.13	47.49	45.83	28.59	>0.05
Postoperative Karnofsky performance status, mean (sd)	76.88	(8.73)	75	(7.56)	78.75	(9.91)	>0.05
Minimum distance to CST, mean (sd)	4.83	(5.86)	5.7	(6.97)	3.96	(4.82)	>0.05
Minimum distance to hand knob, mean (sd)	15.29	(12.63)	16.44	(15.50)	14.13	(9.93)	>0.05
Acute sensorimotor deficit	3	(18.75)	1	(12.50)	2	(25.00)	>0.05
Postoperative PT/OT	11	(68.75)	6	(75.00)	5	(62.50)	>0.05
Baseline CL 9HPT, mean (sd)	46.23	(36.19)	50.05	(37.49)	39.2	(33.38)	>0.05
Baseline IL 9HPT, mean (sd)	27.17	(9.35)	25.75	(4.06)	23.08	(2.91)	>0.05
Change CL 9HPT, mean (sd)	−8.27	(31.64)	−25.66	33.09	9.11	(18.62)	n.c.

*Note:* Total sample size *n* = 16. Glioblastoma = glioblastoma, O‐6‐methylguanine‐DNA methyltransferase = methylguanine‐DNA methyltransferase gene promotor, isocitrate dehydrogenase Wt = isocitrate dehydrogenase wild type, CST = corticospinal tract, CL = contralesional hand, IL = ipsilesional hand.

Abbreviations: 9HPT, nine hold peg test; *n*, number; n.c., not calculated; sd, standard deviation.

### 3.2. Whole‐Brain Sum of SMN Connectivity

We examined the nodal strength of the SMN A (i.e., sum of all connections from the ipsilesional SMN) using a 2‐way mixed model ANOVA and discovered a time × outcome interaction (Figure 2A, *F*_1,12_ = 8.69, $\eta_{p}^{2}$ = 0.42, *p* = 0.01). There was no main effect of time (*F*_1,12_ = 0.88, $\eta_{p}^{2}$ = 0.07, *p* = 0.37) or outcome (*F*_1,12_ = 0.01, $\eta_{p}^{2}$ = 0.001, *p* = 0.94). There were no significant time × scan parameter interactions or main effects of variable scan parameters (*p*  > 0.05).

### 3.3. Homologous Interhemispheric SMN Connectivity

**Figure 3:**
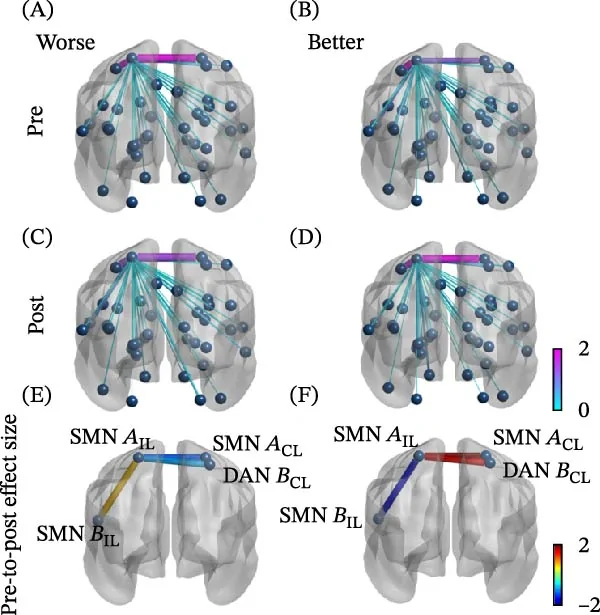
Differences in pairwise network‐to‐network connectivity split by time and outcome. (A, B) Group average Fisher’s *r*‐to‐*z*‐transformed partial correlations at the preresection time point between ipsilesional somatomotor network and other networks of the brain for worse and better recovery, respectively. (C, D) Group average partial connectivity at the follow‐up time point between ipsilesional somatomotor network and other networks of the brain for worse and better recovery, respectively. (E, F) Cohen’s *d* effect size for the pre‐to‐follow‐up difference for worse and better recovery, respectively.

Topographies of networks of interest for pairwise connectivity analyses are presented in Figure 2B. Connectivity Fisher’s *z*‐scores split by outcome and time along with post‐minus‐pre Cohen’s *d* effect sizes are presented in Figure 3. Based on a 2‐way mixed Model ANOVA accounting for variable scan parameters, there was a significant time × outcome interaction for interhemispheric connectivity between the ipsilesional and contralesional SMN A networks (Figure 2C, *F*_1,12_ = 12.57, $\eta_{p}^{2}$ = 0.51, *p* = 0.004). There were no main effects of time (*F*_1,12_ = 0.02, $\eta_{p}^{2}$ = 0.002, *p* = 0.89) or outcome (*F*_1,12_ = 1.12, $\eta_{p}^{2}$ = 0.09, *p* = 0.31). No other comparisons were statistically significant (*p*  > 0.05). There were no significant time × scan parameter interactions or main effects of variable scan parameters (*p*  > 0.05).

### 3.4. Heterologous Intrahemispheric SMN Connectivity

Based on a 2‐way mixed model ANOVA, there was a significant time× outcome interaction for intrahemispheric connectivity between the ipsilesional SMN A and SMN B networks (Figure 2D, *F*_1,12_ = 8.21, $\eta_{p}^{2}$ = 0.41, *p* = 0.014).

Resection in the better recovery group than the worse recovery group (*p* = 0.004). In the better recovery group, connectivity decreased from preresection to follow‐up (*p*  < 0.05). In the worse there were no significant main effects of time (*F*_1,12_ <0.01, $\eta_{p}^{2}$<0.01, *p*  > 0.99) or outcome (*F*_1,12_ = 1.01, $\eta_{p}^{2}$ = 0.08, *p* = 0.34). Voxel size was a significant covariate of ipsilesional SMN A to SMN B connectivity (*F*_1,12_ = 5.17, $\eta_{p}^{2}$ = 0.30, *p* = 0.04). There were no other significant time × scan parameter interactions or main effects of variable scan parameters (*p*  > 0.05).

### 3.5. Heterologous Intrahemispheric Somatomotor to DAN Connectivity

Based on a 2‐way mixed model ANOVA, there was no time × outcome interaction or main effects of outcome or time for ipsilesional SMN A to ipsilesional DAN B networks (Figure 2E, *p*  > 0.05). Thus, hand motor recovery did not appear to largely depend on within‐hemisphere somatomotor to dorsal attention whole‐network plasticity.

### 3.6. Heterologous Interhemispheric Somatomotor to DAN Connectivity

Based on a 2‐way mixed model ANOVA, there was a significant time × outcome interaction for interhemispheric connectivity between the ipsilesional SMN A and contralesional DAN B networks (Figure 2F, *F*_1,12_ = 7.94, $\eta_{p}^{2}$ = 0.40, *p* = 0.017). No other comparisons were statistically significant (*p*  > 0.05). There were no significant time × scan parameter interactions or main effects of variable scan parameters (*p*  > 0.05).

### 3.7. Connectomic Predictors of Dexterity Measured Continuously

**Figure 4:**
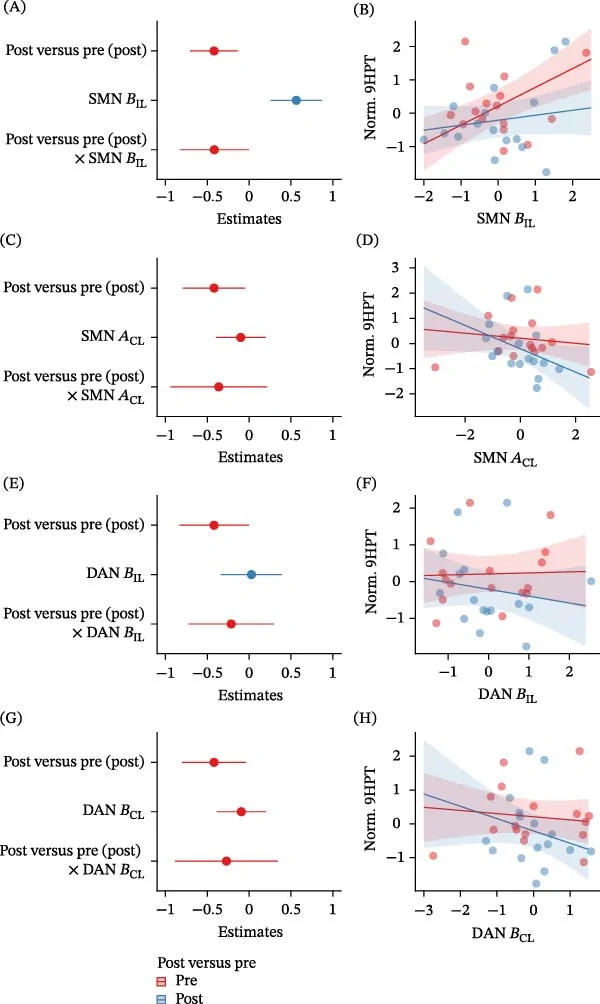
Results of linear mixed effects models of connectomic predictors of dexterity. Panels represent forest plots of magnitudes of linear mixed effects model coefficients with 95%CI and scatter plots presenting marginal effects of the relationship between connectivity and 9HPT at pre‐ and postoperative timepoints. (A, B) Ipsilesional SMN A to ipsilesional SMN B. (C, D) Ipsilesional SMN A to contralesional SMN A. (E, F) Ipsilesional SMN A to ipsilesional DAN B. (G, H) Ipsilesional SMN A to contralesional DAN B.

Multiple linear mixed effects models were computed to predict dexterity on a continuous scale from connectivity (Figure 4). There was no significant interaction effect for time × SMN B (bootstrap *p*_FDR_ = 0.054). There were main effects of time (bootstrap *p*_FDR_ = 0.005) and ipsilesional SMN B connectivity (bootstrap *p*_FDR_ = 0.00056). Figure 4 presents estimates of regression coefficients with false discovery rate adjusted 95% confidence intervals. No other connectivity predictors were significant.

### 3.8. Covariate Controlled Prediction of Follow‐Up Dexterity

**Figure 5:**
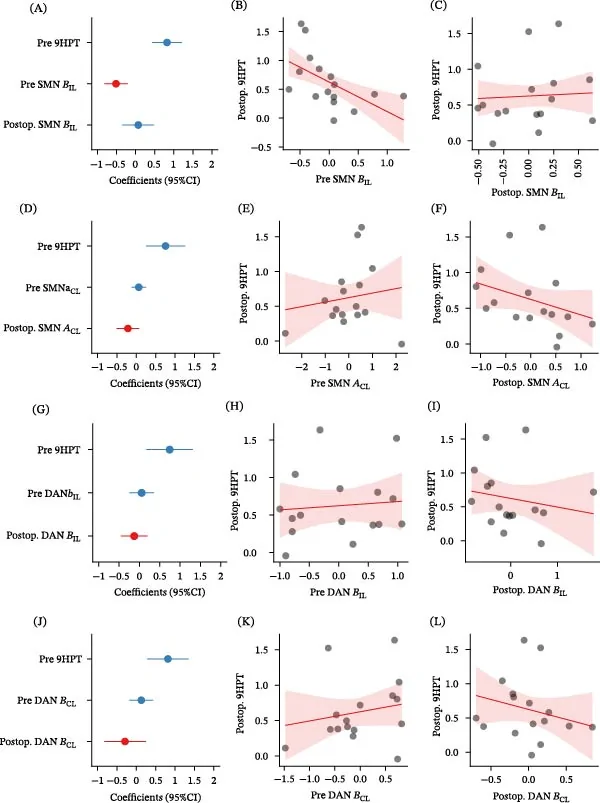
Connectomic predictors across time controlling for preoperative state. Panels represent forest plots of magnitudes of regression coefficients with 95% CI and scatter plots presenting marginal effects of pre‐ and follow‐up connectivity on follow‐up 9HPT while controlling for pre‐9HPT. (A–C) Ipsilesional SMN A to the ipsilesional SMN B. (D–F) Ipsilesional SMN A to contralesional SMN A. (G–I) Ipsilesional SMN A to ipsilesional DAN B. (J–L) Ipsilesional SMN A to contralesional DAN B. Data are from *z*‐score normalized 9HPT followed by transformation to approximate a normal distribution. Higher scores are worse. CL = contralesional, IL = ipsilesional, and Postop. = postoperative.

Multiple linear regressions of dexterity at the follow‐up timepoint controlling for preoperative dexterity were used to assess the predictive ability of preopoerative and follow‐up connectivity. Figure 5 presents results of these linear models with false discovery rate‐adjusted 95% confidence intervals. Preoperative ipsilesional SMN A to ipsilesional SMN B connectivity significantly related to follow‐up dexterity (*p*_FDR_ = 0.005). Additionally, preoperative 9HPT scores significantly predicted follow‐up 9HPT (*p*_FDR_ = 0.004). Models involving other connectivity predictors were not significant.

## 4. Discussion

Our main finding was that ipsilesional SMN A connectivity to the contralesional SMN A, DAN B, and ipsilesional SMN B differentiated better versus worse recovery more robustly than other connections, tumor volumes, or lesional proximity to the corticospinal tract. Additional important findings included: (1) SMN A connectivity to the contralesional DAN B was higher at follow‐up in patients with better outcomes than those with worse outcomes, (2) higher presurgical ipsilesional SMN A to SMN B connectivity was associated with better dexterity postoperatively, and (3) higher preoperative dexterity was also associated with better postoperative dexterity. Importantly, we obtained similar results using a naïve, data‐driven framework in a secondary analysis (Supporting Information 1), reinforcing the importance of these relationships. Such results are also consistent with our previously published finding demonstrating the ability of hand motor cortex to shift posterolaterally into the more classical sensorimotor “face” area in a case of frontal lobe hand motor area invasion by a tumor [11].

### 4.1. Longitudinal Cotrajectories of Hand Function and Brain Connectivity

To our knowledge, this study represents one of the largest case series prospectively assessing the relationship between hand function and brain connectivity before and after surgery in people with contrast‐enhancing brain lesions. Previously, Vassal et al. [53] determined that recovery of support motor syndrome following tumor resection is related to increases in connectivity at follow‐up between the contralesional support motor area and the ipsilesional precentral gyrus. They did not report on quantitative measures of motor impairments other than a dichotomous reporting of motor deficits [53]. Similar to their results, we demonstrated increased connectivity between the ipsilesional SMN A and the contralesional SMN A (which partially contains the support motor area) and DAN B before/after surgery/recovery correlated with hand dexterity.

Most other longitudinal studies examining functional reorganization from pre‐ to postresection have not stratified patients based on changes in function [11–19, 54]. For example, Nenning et al. [54] explored longitudinally distributed changes in connectivity over time. They found an initial decrease in network anomalies at first follow‐up, like our present study, but did not correlate these changes with function. Additionally, Majos et al. [13] documented reductions in ipsilateral motor activation postoperatively in patients with high‐grade gliomas. While they collected measures of paresis and functional status, no analyses discriminating such groups were performed [13]. Several other studies [12, 14–19] determined that spatial representations of primary motor areas moved over time in patients with brain tumors, corroborating the potential for functional reorganization we also observed. Critically, many of these papers [14–17] used data from patients with low‐grade gliomas, which are slower growing than glioblastomas or metastases, thus allowing the brain more opportunity to undergo plastic changes [55]. Within our current study sample, we cannot disentangle the individual impacts of the tumor growth rate, grade, or infiltration on recovery.

### 4.2. Homotopic Somatomotor Connectivity

We found that interhemispheric connectivity was higher at baseline in those with worse outcomes (Figure 2). Previous studies have suggested that low interhemispheric homotopic functional connectivity (compared to healthy controls) within the SMN is related to poorer overall survival [10]. Indeed, similar findings of worse homotopic connectivity in patients with gliomas have been reported in other studies [4, 9, 56–58]. Based on such findings, it would be intuitive to assume that participants with the worse outcomes would have worse baseline connectivity than those with better outcomes. One potential explanation for this discrepancy would be that our participants in the worse recovery group were higher functioning to begin with (i.e., higher function = higher connectivity vs. lower function = lower connectivity [4]). However, baseline 9HPT scores were not different between groups (Table 1). Conversely, we interpret our findings longitudinally as suggesting that progressively worsening connectivity between these regions is associated with worse outcomes.

### 4.3. Implications for Postoperative Care of Patients With Contrast Enhancing Brain Tumors

Recently, the concept of connectivity‐guided neurorehabilitation has gained traction in the neurooncological community [23]. For example, a proof‐of‐concept study used brain areas with abnormal connectivity as targets for repetitive transcranial magnetic stimulation to treat postoperative depression in patients with brain tumors [59]. Our present results suggest that prehabilitation with brain stimulation to facilitate intrahemispheric connectivity between SMN A and SMN B, paired with dexterous exercises, may facilitate better outcomes.

Alternatively, postoperative facilitation of interhemispheric connectivity may also improve outcomes. One recent study examining repetitive transcranial magnetic stimulation to the interhemispheric SMN A connection failed to show improvements in hand function [25]. It is important to note that Engelhardt et al. [25] applied a generally inhibitory stimulus consistent with stroke studies showing that inhibiting interhemispheric connectivity improves function [60]. Our findings, on the other hand, suggest that a postoperative faciliatory stimulus to the contralesional motor cortex may be necessary. We hypothesize that the differing rates of damage to the cortex between stroke and brain tumors alter how the cortex reorganizes [55, 61].

In sum, our data supports the idea that preoperative facilitation of ipsilesional SMN A to ipsilesional SMN B connectivity may improve outcomes; however, given the urgent need for surgical resection in many cases, this may not be feasible. Alternatively, postoperative enhancement of inter‐regional synchrony (e.g., paired‐pulse transcranial magnetic stimulation, dual‐site transcranial alternating current stimulation, etc.) may be more feasible. Furthermore, since exercise has been shown to be safe and prolongs survival in patients with gliomas [62–64], future studies can examine how exercise impacts interhemispheric connectivity on its own, what its potential role in promoting motor recovery might be after surgery, and how this effect may or may not synergistically align with targeted neuromodulation.

### 4.4. Limitations

While this study represents one of the largest studies to date longitudinally mapping changes in connectivity to changes in hand function, the sample size of 16 limits subgroup analyses. For example, this study was likely underpowered to account for potential covariates such as tumor type, grade, growth rates, and infiltration. Likewise, our study was also likely underpowered to conduct a mediation analysis for the hypothesized path of left‐side tumor → SMN A homotopic connectivity → hand functional outcomes, which, based on a power analysis using our results, would require a minimum of 45 participants. Nonetheless, results from this study can inform the design of larger longitudinal studies to examine these domains, which will be the focus of our future work. Finally, due to variations in clinical protocols, our scan parameters varied across participants. Despite this limitation, our findings were statistically independent of this variability.

## 5. Conclusion

Here, we demonstrate that interhemispheric, homotopic SMN A connectivity differentiated better versus worse recovery of dexterity after recovery from brain tumor surgery. Additional important findings included: (1) SMN A connectivity to the contralesional DAN B was higher at follow‐up in patients with better outcomes than those with worse outcomes, (2) higher presurgical ipsilesional SMN A to SMN B connectivity was associated with better dexterity postoperatively, and (3) higher preoperative dexterity was also associated with better postoperative dexterity. These findings suggest potential targets for neuromodulation‐based rehabilitation therapy to improve hand dexterity outcomes.

## Funding

This project was funded in part by the Advancing a Healthier Wisconsin Endowment (Grant FP00025837).

## Conflicts of Interest

The authors declare no conflicts of interest.

## Supporting Information

Additional supporting information can be found online in the Supporting Information section.

## Supporting information


**Supporting Information** The supplement contains additional secondary analyses conducted on our data.

## Data Availability

All data will be available upon reasonable request to the corresponding author.
