# Supplementary material for: Changes in Somatomotor Network Connectivity Differentiate Better Versus Worse Recovery of Hand Dexterity After Brain Tumor Surgery: A Cohort Study
Source: Neural Plast. 2026 Sep 21;2026:3255120. doi: 10.1155/np/3255120 (PMC13594770; doi:10.1155/np/3255120)
Supplement: Supplementary file 1 [file NP-2026-3255120-s001.docx]

**Supplementary Materials**

Demographic variables were examined for effects on our pre- vs post- surgery connectivity using a 2 by 2 mixed model ANCOVA. There were significant age-by-time interactions for connectivity to the contralesional SMN A (F_1,14_=6.13, $\eta_{p}^{2}$=0.30, p=0.03), but not other variables of interest. There were also Left-sided tumor by time interaction for connectivity to the contralesional SMN A (F_1,14_=6.05, $\eta_{p}^{2}$=0.30, p=0.03); however, after Sidak correction, there were no significant pairwise comparisons. There was also a left-side tumor-by-time interaction for connectivity to the contralesional DAN B (F_1,14_=12.30, $\eta_{p}^{2}$=0.47, p=0.003), but not to other variables of interest. Finally, there was a main effect of biological sex on SMN A node strength (F_1,14_=5.37, $\eta_{p}^{2}$=0.28, p=0.04). Females, overall, had higher SMN A node strengths compared to males. Notably, extent of resection and distance between tumor to the hand knob, SMN A, or corticospinal tract did not impact connectivity (all p>0.05).

Secondary to our finding that homotopic SMN A connectivity was more strongly associated with outcome than ipsilesional SMN A node strength, we sought to understand what clinical variables would best predict change in homotopic SMN A connectivity over time. Predictor variables in univariate analyses included left vs. right side tumor location, glioblastoma vs non-glioblastoma, age, male vs female, post operative restricted diffusion (i.e. infarct), and tumor proximity to hand knob. Of these, only left-sided tumor (β= -1.61, R^2^=0.55, F_1,14_=6.05, p=0.027) significantly predicted change in homotopic connectivity. All other predictors had p>0.05. Left-sided tumors were significantly more likely to have a better outcome than right-sided tumors (OR=9.60, p=0.007)

Additionally, we examined whether chemotherapy or radiation history may have impacted functional outcomes. No participants were actively receiving radiation at time of follow up. Thirteen of 16 participants completed radiation prior to follow up. Three did not undergo radiation therapy. Two of these participants were the participants with radionecrosis. Twelve of 16 participants were on chemotherapy at time of follow up and 14 total participants received chemotherapy at some point between surgery and follow up. Being on chemotherapy or radiation at follow up did not relate to outcome (p>0.05). Time from start of chemotherapy (Better Recovery = 109.14±28.79, Worse Recovery = 109.71±47.76 days; p>0.05) and radiation (Better Recovery = 114.14±68.08, Worse Recovery = 94.5±39.66 days; p>0.05) to follow up were not different.

Data-Driven Approach

Analytical Methods for Data-Driven Approach

Following our hypothesis-driven analysis, we conducted a data-driven analysis. Initially, pre-resection connectivity values were subtracted from their corresponding postoperative follow-up values for each of the 39 connections from the ipsilesional SMN A to the other regions of interest. These connectome change scores were then entered into a k-means clustering algorithm in MATLAB to determine data-driven patterns of recovery. To accomplish this, first we used the *evalclusters* function to identify the optimal *k* for use in k-means clustering. This relied upon the Calinski-Harabasz criterion, and possible values for *k* from *k*=1 to *k*=16 were evaluated. This resulted in an optimal *k*=2. *K*-means clustering was then undertaken on the change in connectivity scores with *k*=2 in order to minimize the cosine distance with cluster centers.^65,66^ Clustering was replicated 10,000 times to ensure stability of results, as clustering initiates with a random starting point. Upon identifying clusters, a linear classifier (*fitclinear)* was used to identify features (i.e. variables) that were most important for predicting cluster assignment. For this analysis, we used a leave-one-out cross validation with L1 (least absolute shrinkage and selection operator [LASSO]) regularization, and coefficients were averaged across cross-validation partitions.

Cluster assignments were then used as a grouping variable for a 2-way mixed model ANOVA for 9HPT over time (pre-resection to post-resection follow-up). That is, 9HPT score (in seconds) was the dependent variable, and cluster assignment and time (pre-resection to post-resection follow-up) were the between and within group factors, respectively. As such, this analysis represents a linear assessment of the relationship between patterns of change in connectivity and change in 9HPT scores. 9HPT scores (seconds) were natural log transformed to correct for a rightward skew. Additionally, cluster assignment was used in a receiver operator characteristic (ROC) analysis (SPSS) to predict Good vs. Poor recovery status (that is, the outcome factor from the above analyses which was based on being above or below median change in 9HPT time) from the hypothesis-driven framework. Logistic regressions were additionally used to predict cluster assignment from demographic data. As with the hypothesis-driven framework, alpha was set at p<0.05.

Results of our Data Driven Approach

Participants’ changes in connectivity from pre-resection to post-operative follow-up were clustered to resolve a data-driven separation of connectivity outcomes naïve to functional outcomes. The *k­*-means clustering assigned 9 participants to Cluster 1 and 7 participants to Cluster 2. The strongest predictor of cluster assignment was the ipsilesional SMN A to contralesional SMN A. This was then used to compare changes in log_10_-transformed 9HPT times. Using a mixed model ANOVA, there was a significant Cluster by time interaction (Fig 5a-b, F_1,14_=4.70, $\eta_{p}^{2}$=0.25, p=0.048). There was a significant improvement in 9HPT times for Cluster 2 (p=0.035) but no change in 9HPT times for Cluster 1 (p=0.48). There were no main effects of Cluster ID or time on 9HPT scores (p>0.05). Similarly, Cluster IDs were able to discriminate good vs. poor recovery based on change of 9HPT time (Fig 5b, Area under the curve=0.88, p<0.001). Weights for contributions to cluster assignment for all connections are displayed in Fig 6. Univariate logistic regressions were performed to predict Cluster assignments from

Supplemental Figure 1. Clustering of changes of connectivity over time relate to hand functional outcomes in patient with brain tumors. A) Results of a mixed model ANOVA whereby displaying the effects of Cluster assignment and time on natural log transformed 9HPT times. B) Results of change in 9HPT times from pre-resection to follow-up separated by Cluster assignment. This is for visualization only

demographic data. As with the hypothesis-driven framework, participants with left-sided tumors were less likely to be in Cluster 2 (β=3.89, OR=49.00, Nagelkerke R^2^=0.63, p=0.01). None of the other demographic variables were significant predictors of Cluster assignment (p>0.05).

To determine whether pre- vs post-surgery 9HPT scores depended on pre-resection connectivity, we conducted a separate cluster analysis of pre-resection connectivity related to longitudinal changes in dexterity. There was no Cluster by Time interaction (F_1,14_=0.40, $\eta_{p}^{2}$=0.03, p=0.54), main effect of time (F_1,14_=1.13, $\eta_{p}^{2}$=0.08, p=0.31), or main effect of cluster (F_1,14_=0.46, $\eta_{p}^{2}$=0.03, p=0.51).

Supplemental Figure 2. Features most important for discriminating cluster assignment. MATLAB *fitclinear* was used to predict cluster assignment with leave-one-out cross-validation and LASSO regularized coefficients. Cereb=cerebellum, ECN=executive control network, DAN=dorsal attention network, DMN=default mode network, LIM=limbic network. SAL=salience network, SMN=somatomotor network, BG=basal ganglia, TP=temporoparietal network, Thalamus, VCen=visual central network, VPer=Visual peripheral network

Supplementary Figure 3. Tumor lesion density map for better (blue-green) and worse (dark red-yellow) outcome groups.

Worse Outcomes Lesion Density

Better Outcomes Lesion Density

Supplementary Figure 4. Edema lesion density map for better (blue-green) and worse (dark red-yellow) outcome groups.
